# Association between plasma interleukin-33 level and acute exacerbation of chronic obstructive pulmonary disease

**DOI:** 10.1186/s12890-021-01423-8

**Published:** 2021-03-15

**Authors:** Hyonsoo Joo, Seoung Ju Park, Kyung Hoon Min, Chin Kook Rhee

**Affiliations:** 1 Division of Pulmonary, Allergy and Critical Care Medicine, Department of Internal Medicine, Uijeongbu St. Mary’s Hospital, College of Medicine, The Catholic University of Korea, Seoul, Republic of Korea; 2 Division of Pulmonary, Allergy and Critical Care Medicine, Department of Internal Medicine, Jeonbuk National University Hospital, Jeonbuk National University Medical School, Jeonju, Republic of Korea; 3 Division of Pulmonary, Allergy, and Critical Care Medicine, Department of Internal Medicine, Korea University Guro Hospital, Korea University College of Medicine, 148, Gurodong-ro, Guro-gu, Seoul, 08308 Republic of Korea; 4 Division of Pulmonary, Allergy and Critical Care Medicine, Department of Internal Medicine, Seoul St. Mary’s Hospital, College of Medicine, The Catholic University of Korea, 222 Banpodaero, Seochogu, Seoul, 06591 Republic of Korea

**Keywords:** Interleukin-33, Chronic obstructive pulmonary disease, Exacerbation

## Abstract

**Background:**

The role of interleukin (IL)-33 in patients with chronic obstructive pulmonary disease (COPD) has not been well elucidated. The aim of this study is to analyze the association between plasma IL-33 level and acute exacerbation of COPD.

**Methods:**

Plasma IL-33 was measured in 62 COPD patients during their stable state. Patients were prospectively followed up for 1 year. The expression of IL-33 was measured in lung tissue obtained from 38 patients who underwent surgery.

**Results:**

The number of exacerbations was significantly higher in the high plasma IL-33 group compared with the low plasma IL-33 group. On Poisson regression analysis, high plasma IL-33 was associated with increased risk of exacerbation (incidence rate ratio = 2.166, *P* = 0.043). The expression of IL-33 in the lung was higher in COPD patients than in controls. The expression of IL-33 was significantly correlated with smoking pack years (R = 0.45, *P* < 0.01) and Forced expiratory volume in 1 s (%) (R = − 0.58, *P* < 0.01).

**Conclusion:**

The plasma level of IL-33 in patients with COPD was significantly associated with the risk of exacerbation in prospective follow up. The expression of IL-33 in the lung was positively correlated with smoking and negatively correlated with lung function.

## Introduction

Interleukin (IL)-33, classified as an IL-1 family, is an important cytokine involved in type 2 immunity and systemic inflammation. IL-33 is highly expressed in bronchial epithelial cells of asthma [1]. The IL-33/ST2 receptor complex signal pathway via group 2 innate lymphoid cells (ILC2) has been suggested to play a major role in the pathogenesis of asthma in humans. IL-33 promotes the ILC2 response, which secretes a large amount of IL-5 and IL-13 in allergic inflammation in asthma patients [2]. Increased expression of IL-33 according to asthma severity in human has been reported in several studies [3, 4]. On the other hand, cigarette smoke (CS) can also induce IL-33 production. The expression levels of IL-33 and ST2 were markedly enhanced in the lung tissue of mice inhaling cigarette smoke [5].

The role of IL-33 in patients with chronic obstructive pulmonary disease (COPD) has not been well elucidated compared to that in patients with asthma. Xia et al. [6] reported that the plasma level of IL-33 was significantly higher in COPD patients than in controls. Gorska and colleagues reported that the levels of IL-33 in serum, induced sputum, exhaled breath condensate, and bronchial mucosa were similar between asthma and COPD [7]. However, whether IL-33 levels in COPD patients are associated with acute exacerbation has not yet been evaluated. The aim of this study is to analyze the association between plasma IL-33 levels and COPD acute exacerbation.

## Material and methods

### Study design

Plasma IL-33 levels were measured in patients enrolled in prospective cohort. To develop optimal clinical and biological markers for COPD, the chronic airway disease cohort study was performed. Biomarker studies using this cohort have already been published [8–11]. Patients were enrolled at Seoul St. Mary’s Hospital, Chonbuk National University Hospital, and Korea University Guro Hospital between June 2015 and July 2017. In total, 355 patients with chronic airway disease were enrolled. Among them, patients with COPD were analyzed in this study. The criteria for COPD were as follows: (1) age ≥ 40 years, (2) post-bronchodilator forced expiratory volume in 1 s (FEV_1_)/forced vital capacity (FVC) < 0.7, and (3) history of smoking (pack-years ≥ 10). At enrollment, baseline characteristics including age, sex, body mass index (BMI), smoking history, comorbidities, and COPD medication were collected. Pulmonary function test (PFT), fractional exhaled nitric oxide (FeNO), complete blood count, and blood chemistry were measured. COPD assessment test (CAT), and Modified Medical Research Council (mMRC) dyspnea scale were also surveyed. Blood and urine samples were collected at enrollment and stored. Patients were prospectively followed for 1 year and monitored for exacerbation. PFT was performed one year after the enrollment. A total of 62 plasma samples obtained from COPD patients were analyzed in this study.

A total of 38 lung tissue samples were obtained from Korea Guro Hospital Biobank. Patients underwent surgery between January 2008 and January 2015 due to of lung cancer. Baseline characteristics and clinical information were obtained by retrospective review. Eleven patients were never smokers, nine were smokers without airflow obstruction, and eighteen were COPD. There was no patient with other chronic pulmonary disease such as asthma or interstitial lung disease.

This study was approved by the Institutional Review Board of Seoul St. Mary’s Hospital (KC15OIMI0553), Chonbuk National University Hospital (2015-01-018-005), and Korea University Guro Hospital (KUGH 13246). Written informed consent was provided by all patients.

### Plasma IL-33 measurement

Plasma IL-33 level was measured using a technique described previously [12]. Briefly, the blood samples were centrifuged (10 min at 1,000×*g*) within 30 min after collection in tubes containing sodium ethylenediaminetetraacetic acid anticoagulant, and the collected plasma was stored at ≤ − 20 °C. Plasma samples were prepared for analysis in a 96-well plate utilizing a custom human cytokine. IL-33 level was measured by enzyme-linked immunosorbent assay (ELISA) using a Human IL-33 Quantikine ELISA Kit (R&D Systems, Minneapolis, MN, USA) and 12 times concentrated with Amicon Ultra-0.5 mL Centrifugal Filter (Merck Millipore, Burlington, MA, USA) devices to improve the detection yield. We followed the kit-specific protocol provided with the BioTek’s PowerWave XS analyser (BioTek Instruments, Winooski, VT, USA). The result is described as 450 optical density (OD).

### Histologic analysis

For each 38 patients, 5 slides were prepared by the Biobank of Korea University Guro Hospital; 1 with Mariendfeld 76 × 26 superior slide for Hematoxylin eosin (H&E) stain and 3 slides with MUTO New silane III for immunohistochemical stain. Immunohistochemical studies were performed on 4-μm-thick tissue sections by using a Bond 3 automated immunostainer (Leica Microsystems, Wetzlar, Germany) with primary antibodies against IL-33 (R&D Systems). The immunohistochemical staining protocol for paraffin-embedded specimens was as follows; sample preparation, deparaffinization/rehydration, antigen retrieval, staining, and dehydrating and mounting sections. To wash out paraffin, the slides were placed in three containers of xylene for 5 min each. For rehydration, slides were placed in containers containing 100%, 90%, 80%, and 70% ethanol for 1 min each to remove xylene. The slides were washed with dH_2_O to complete the rehydration process. Next, antigen retrieval was conducted by heating the sections in a microwave for 10 min with sodium citrate buffer at a pH 9.0 for detecting IL-33. To quench endogenous peroxidase activity in samples, sections were placed in 3% hydrogen peroxide for 10 min. To prevent non-specific binding of the antibody to the tissues, each section was blocked with 100–400 μL of blocking solution for 1 h at room temperature in a humidified chamber for 20 min. Diluted primary antibodies were dropped and incubated for 1–2 h at room temperature. They were washed with Tris-buffered saline three times. The secondary antibody was incubated for 20–30 min and also washed with Tris-buffered saline three times. Counterstain sections with hematoxylin were used. This stains the blue cell nuclei, which provides a contrast to the brown color of the 3,3′-diaminobenzidine chromogen for better visualization of tissue morphology. All of these steps were in automated process and the slides were pulled out from the Bond 3 automated immunostainer and dehydrated manually with 70%, 80%, 90%, and 100% ethanol again. The coverslips were mounted and set and the slides were set to view on a microscope.

Immunostaining scoring was carried out by the GenASIs capture and analysis system (ADS BIOTEC, Omaha, NE, USA). This program is capable of accurately match digitally annotated H&E sections with tissue on scanned immunohistochemistry slides. H&E slides were scanned on the Aperio AT scan scope (Leica Biosystems, Wetzlar, Germany) at × 20 magnification and the thumbnail images were imported into the GenASIs sytem. The images were then aligned using the GenASIs software. The GenASIs Spotscan is able to provide statistical analysis of all cell counts, showing user-defined cell clusters based on signal counts. The program also detects and enumerates small and faint signals. The IL-33 positive stain cell percentage per slide was measured in the same length (120 μm) of airway epithelium.

### Statistical analysis

Student’s t-test was used for normally distributed data and Mann–Whitney U-test was used for non-normally distributed data to compare continuous variables between two groups. Analysis of variance (ANOVA) was used to compare continuous variables between four groups. The chi-square and Fisher’s exact tests were used to compare categorical variables. Correlations between two groups were analyzed by Pearson’s correlation coefficients. Poisson regression analysis was performed to compare exacerbation rate. Statistical significance was defined as *P* < 0.05. All analyses were performed using SPSS Statistics for Windows version 22.0 software (IBM, Armonk, NY, USA).

## Results

### Baseline characteristics

Baseline characteristics of enrolled patients are described in Table 1. Among 62 patients with stable COPD, 98.4% were male and the mean age was 65.1 years. About half patients were current smoker and the average number of pack years was 44.1. All patients had a history of smoking greater or equal to 10 pack years. The mean FEV_1_ was 59.2% and the mean CAT score was 14.7. All patients were prescribed long-acting bronchodilators with/without inhaled corticosteroid.

**Table 1:** Baseline characteristics of patients with COPD in the cohort

Characteristics	Total (n = 62)	Low IL-33 (n = 45)	High IL-33 (n = 17)	*P* value
Age (years)	65.1 ± 9.0	66.9 ± 8.8	60.1 ± 7.8	0.01
Male	61 (98.4%)	44 (97.8%)	17 (100.0%)	1.00
Smoking status				
Current smoker	30 (48.4%)	25 (55.6%)	5 (29.4%)	0.09
Ex-smoker	32 (51.6%)	20 (44.4%)	12 (70.6%)	
Pack years	44.1 ± 21.5	45.8 ± 19.8	39.7 ± 25.7	0.17
BMI (kg/m^2^)	23.2 ± 3.4	23.3 ± 3.3	22.9 ± 3.8	0.88
PFT				
Post BD FVC (L)	3.51 ± 0.73	3.45 ± 0.74	3.68 ± 0.68	0.42
Post BD FVC (%)	82.1 ± 15.7	81.8 ± 17.5	82.9 ± 9.9	0.94
Post BD FEV_1_ (L)	1.86 ± 0.68	1.84 ± 0.65	1.92 ± 0.78	0.62
Post BD FEV_1_ (%)	59.2 ± 17.8	60.2 ± 17.2	56.6 ± 19.5	0.55
Post BD FEV_1_/FVC (%)	51.9 ± 13.3	52.4 ± 12.8	50.6 ± 15.0	0.80
Symptom				
CAT	14.7 ± 7.2	15.2 ± 7.5	13.2 ± 6.2	0.37
mMRC	0.8 ± 0.9	0.8 ± 0.9	0.8 ± 0.9	0.89
Blood eosinophil count (/μl)	257.4 ± 242.6	230.9 ± 228.0	327.3 ± 272.4	0.16
Number of exacerbations/yr	0.56 ± 0.95	0.40 ± 0.62	1.00 ± 1.46	0.01
IL-33 (450 OD)	0.069 [0.065–0.075]	0.067 [0.064–0.070]	0.079 [0.076–0.100]	< 0.01
COPD medication				
LABA	2 (3.2%)	2 (4.4%)	0 (0.0%)	1.00
LAMA	5 (8.1%)	5 (11.1%)	0 (0.0%)	0.31
LABALAMA	12 (19.4%)	11 (24.4%)	1 (5.9%)	0.15
ICSLABA	6 (9.7%)	3 (6.7%)	3 (17.6%)	0.33
ICSLABALAMA	38 (61.3%)	25 (55.6%)	13 (76.5%)	0.16

Data are expressed as mean ± SD or median [IQR] or No. (%)

COPD, chronic obstructive pulmonary disease; IL, interleukin; BMI, body mass index; PFT, pulmonary function test; BD, bronchodilator; FVC, functional vital capacity; FEV_1_, forced expiratory volume in 1 s; CAT, COPD assessment test; mMRC, modified Medical Research Council; yr, year; OD, optical density; LABA, long acting beta agonist; LAMA, long acting muscarinic antagonist; ICS, inhaled corticosteroid

### Comparison between high and low plasma IL-33 groups

**Fig. 1:**
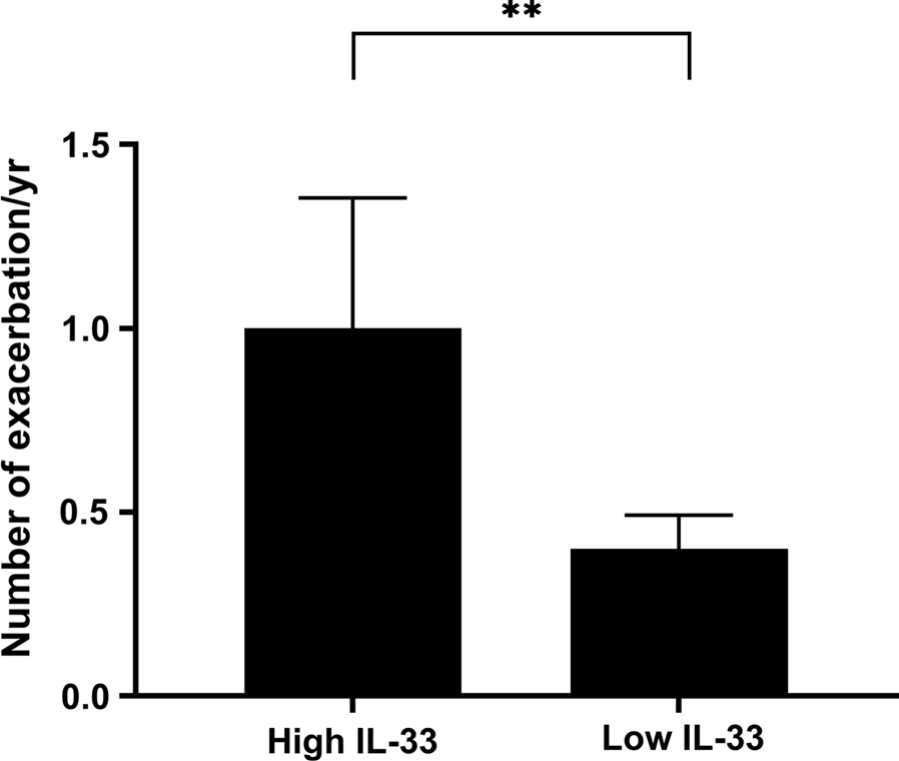
Comparison of the number of exacerbations/year according to plasma IL-33 level. **P < 0.01. Abbreviations: IL, interleukin

Patients were grouped into high and low IL-33 group. Patients with highest quartile level of IL-33 (n = 17) were classified into the high group. The other 45 patients were in the low group. The mean age in the high IL-33 group was significantly lower than that in the low IL-33 group (60.1 vs 66.9, *P* = 0.01). There were no significant differences in other variables. During the prospective follow-up period of one year, the mean number of exacerbations was 0.56 ± 0.95. The number of exacerbations was significantly higher in the high IL-33 group compared with the low IL-33 group (1.00 ± 1.16 vs 0.40 ± 0.62, *P* = 0.01; Fig. 1).

**Fig. 2:**
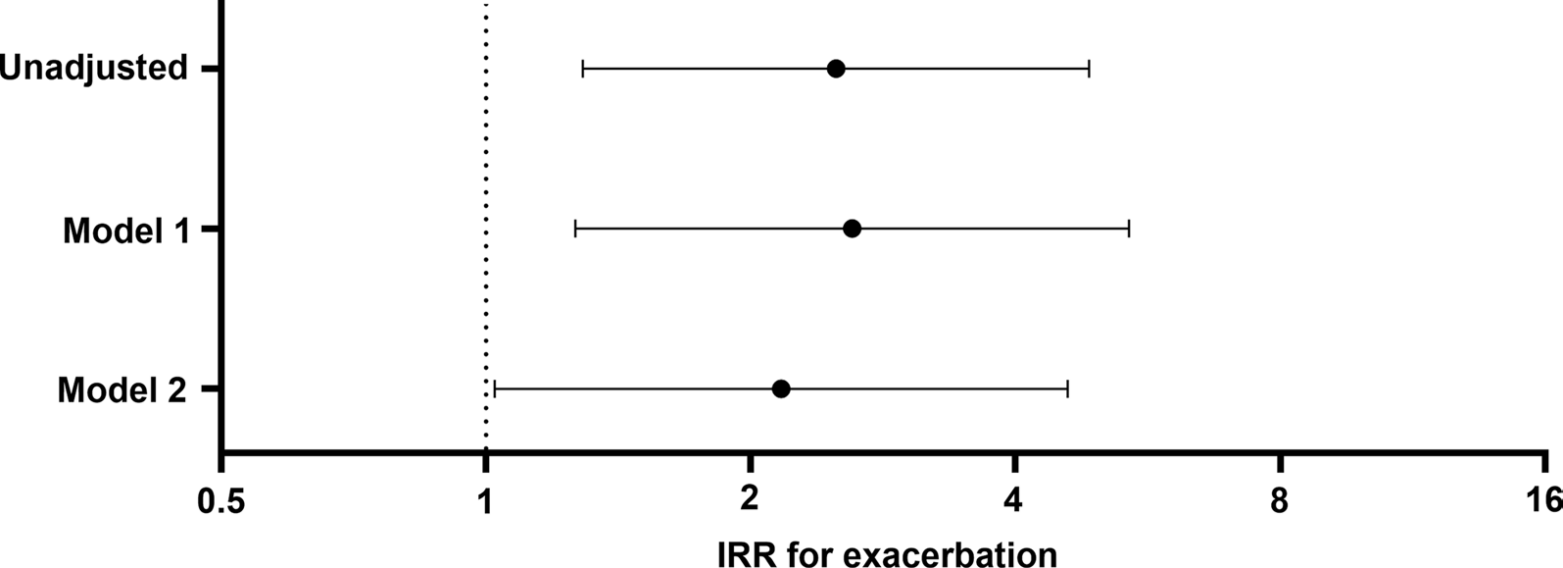
Risk of exacerbation in patients with high plasma IL = 33. Model 1: adjusted by age and sex, Model 2: adjusted by age, sex, and FEV_1_ (%). Abbreviations: IRR, incidence rate ratio; IL, interleukin; FEV_1_, forced expiratory volume in 1 s

In the unadjusted model, the incidence rate ratio (IRR) of the high IL-33 group for exacerbation was 2.500 (95% confidence interval [CI]: 1.288–4.851, *P* = 0.01, Fig. 2). The IRR for model 1 (adjusted by age and sex) was 2.608 (95% CI: 1.263–5.382, *P* = 0.01) and model 2 (adjusted by age, sex, and FEV_1_ (%)) was 2.166 (95% CI: 1.023–4.584, *P* = 0.043, Table 2).

**Table 2:** Poisson regression analysis for the risk of exacerbation

Variables	IRR	95% CI	*P* value
Age	0.984	0.941–1.030	0.500
Female	5.956	0.623–56.914	0.121
Post BD FEV_1_ (%)	0.979	0.961–0.998	0.032
High IL-33	2.166	1.023–4.584	0.043

IRR, incidence rate ratio; CI, confidence interval; BD, bronchodilator; FEV_1_, forced expiratory volume in 1 s; IL, interleukin

### Human lung tissue analysis

**Fig. 3:**
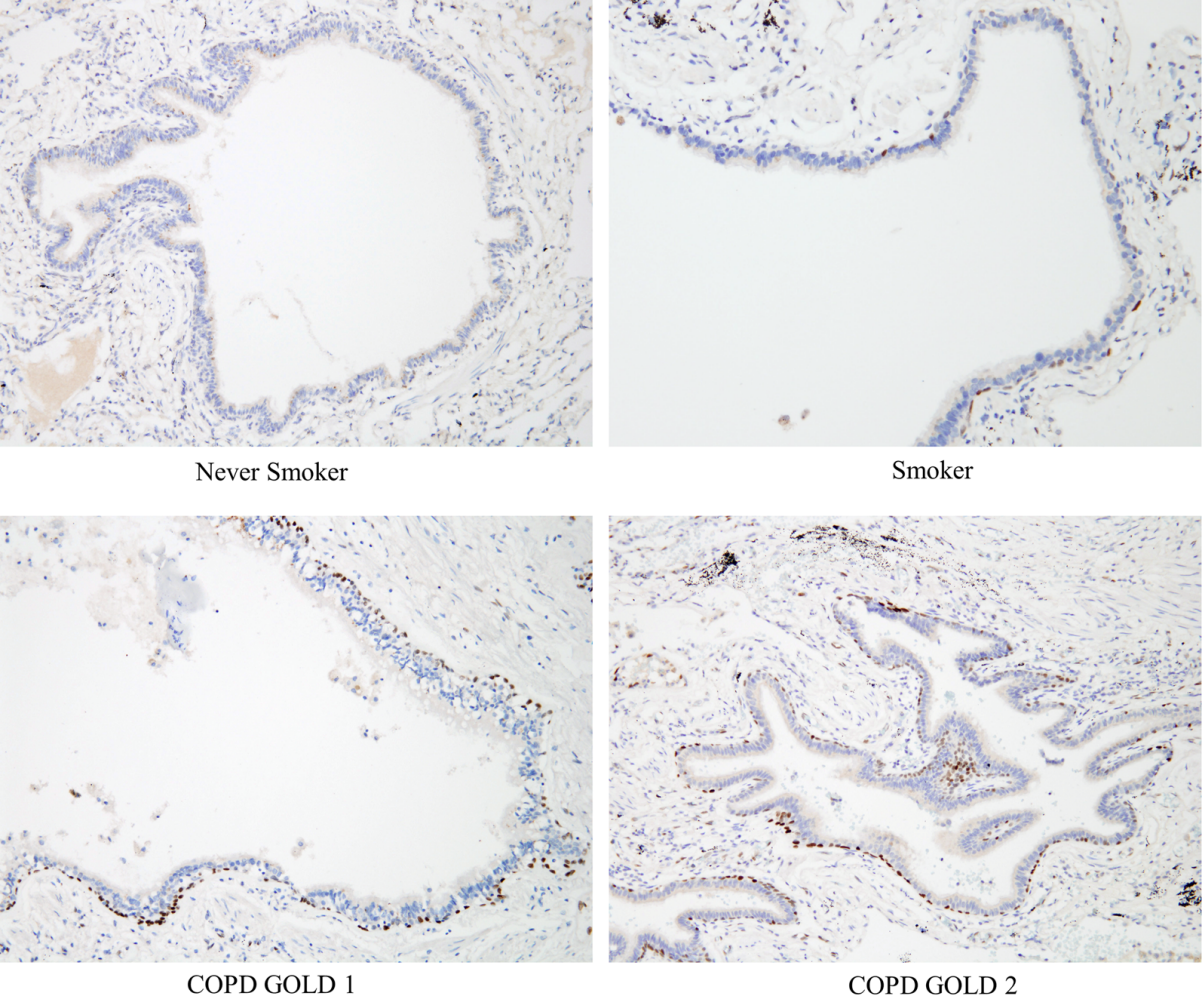
IHC analysis of IL-33 in human lung tissue. Abbreviations: IHC, immunohistochemistry; IL, interleukin

**Fig. 4:**
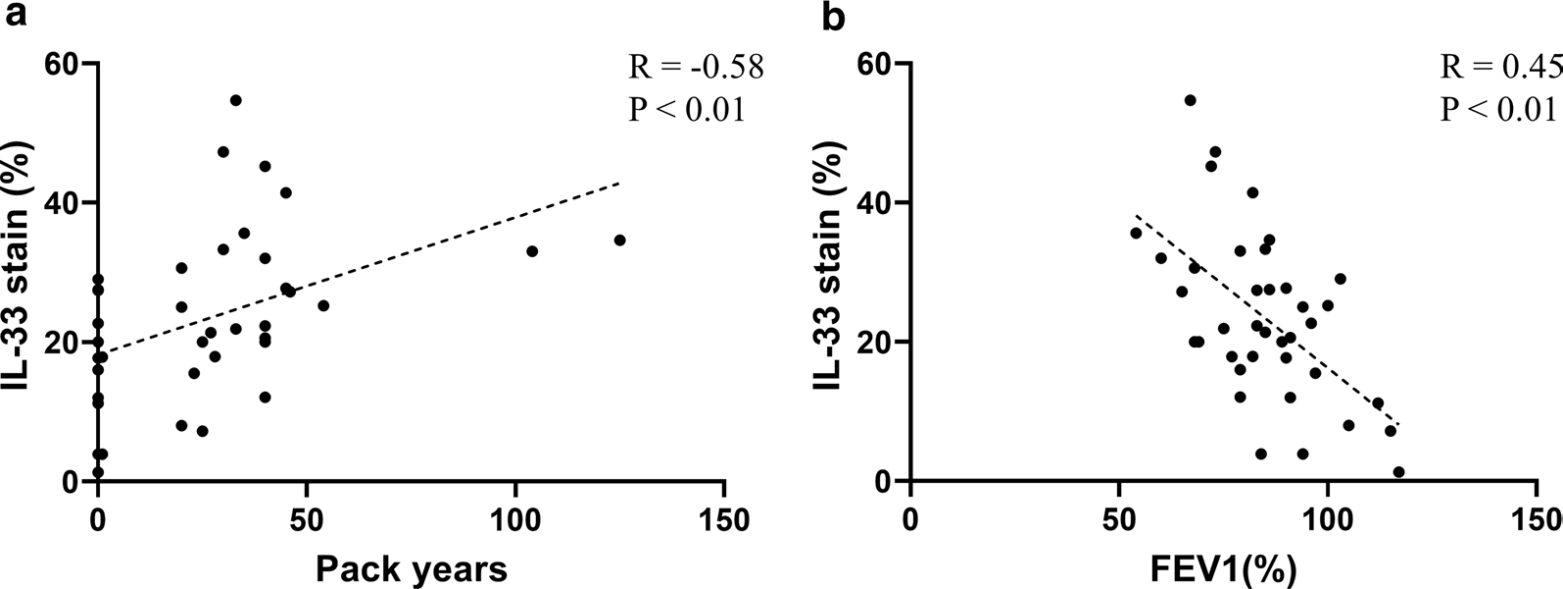
Correlation between the expression of IL-33 in lung tissue and amount of smoking (**a**). Correlation between IL-33 and FEV_1_ (%) (**b**). Abbreviations: R, Pearson correlation coefficient; IL, interleukin; FEV_1_, forced expiratory volume in 1 s

The baseline characteristics of patients with human lung tissue are listed in Table 3. Patients were divided into 4 groups according to their smoking history and lung function. The FEV_1_ (%) of all patients was over 50%. This is because the patients with low lung function could not undergo surgery due to the risk of complications. Age, sex, smoking status, and PFT differed significantly between groups. The mean age was the lowest in smoker group. Males were predominant in the smoker/COPD group, whereas females were more common in the group that had never smoked. The expression of IL-33 differed significantly between groups (Fig. 3). The expression of IL-33 was lowest in never smoker and highest in Global Initiative for Chronic Obstructive Lung Disease (GOLD) stage 2 COPD patients (Table 3). There was significant correlation between IL-33 expression and smoking pack years (R = 0.45, *P* < 0.01; Fig. 4). Also, IL-33 expression was significantly inversely correlated with FEV_1_ (%) (R = − 0.58, *P* < 0.01).

**Table 3:** IL-33 Baseline characteristics of patients with lung tissue analysis

Characteristics	Never smoker	Smoker	GOLD 1	GOLD 2	*P* value
	(n = 11)	(n = 9)	(n = 8)	(n = 10)	
Age (years)	65.5 ± 8.6	57.9 ± 8.9	63.1 ± 7.5	66.9 ± 5.6	0.08
Male	2 (18.2%)	8 (88.9%)	8 (100.0%)	10 (100.0%)	< 0.01
Smoking status					
Current smoker	0 (0.0%)	2 (22.2%)	1 (12.5%)	3 (30.0%)	< 0.01
Ex-smoker	0 (0.0%)	7 (77.8%)	7 (87.5%)	7 (70.0%)	
Pack years	0.0 ± 0.0	37.9 ± 35.7	31.0 ± 15.1	42.1 ± 22.9	< 0.01
PFT					
Post BD FVC (L)	2.48 ± 0.4	3.72 ± 0.9	4.30 ± 0.5	3.59 ± 0.7	< 0.01
Post BD FVC (%)	85.6 ± 10.4	84.9 ± 18.2	100.4 ± 7.4	84.6 ± 10.0	0.03
Post BD FEV_1_ (L)	2.00 ± 0.4	3.01 ± 0.6	2.76 ± 0.3	2.10 ± 0.3	< 0.01
Post BD FEV_1_ (%)	91.8 ± 14.3	91.2 ± 14.7	87.8 ± 4.7	69.2 ± 8.1	< 0.01
Post BD FEV_1_/FVC (%)	80.6 ± 6.9	82.0 ± 6.6	64.1 ± 4.9	59.6 ± 10.0	< 0.01
RV (%)	102.9 ± 26.5	100.3 ± 30.1	113.1 ± 18.0	121.4 ± 25.6	0.32
IL-33 stain (%)	17.2 ± 9.4	18.5 ± 8.4	24.4 ± 10.9	34.0 ± 12.6	< 0.01

Data are expressed as mean ± SD or No. (%)

GOLD, global initiative for chronic lung disease; PFT, pulmonary function test; BD, bronchodilator; FVC, forced viral capacity; FEV_1_, forced expiratory volume in 1 s, RV, residual volume; IL, interleukin

## Discussion

Only a few studies have investigated the role of IL-33 in patients with COPD. There are controversial results regarding whether IL-33 expression is increased in COPD. Tang et al. [13] examined the roles of serum IL-33 by ELISA in COPD patients and found that the levels of serum IL-33 in COPD patients were lower than in health controls. However, Xia and colleagues [6] showed that serum IL-33 level measured by ELISA was elevated in patients with COPD compared with controls. Also, the expression of IL-33 in lungs of COPD patients was higher than in controls. Tworek et al. [14] measured IL-33 level by ELISA in exhaled breath condensate. IL-33 was higher in COPD compared with healthy non-smokers. Kearley and colleagues [15] showed that IL-33 concentrations measured in lung tissue homogenate by bead-based immunoassay were higher in GOLD 3-4 COPD compared with controls.

The results of our study are compatible with those of Xia [6], Tworek [14], and Kearley [15]. In lung tissue analysis, the expression level of IL-33 was higher in COPD patients than in controls. Interestingly, we have shown that the level of IL-33 expression was well correlated with the amount of smoking. This result suggests that smoking may induce IL-33 expression in the lungs and contribute to the progression of COPD. Lung function was negatively correlated with the IL-33 expression in lung tissue. Kearley et al. [15] also showed that IL-33 expression in lung is inversely correlated with FEV_1_ (%) in patient with COPD.

All previous studies [6, 14, 15] except one [13] showed that IL-33 was higher in COPD compared with controls. There can be a potential reason for the discrepant result of study by Tang et al. [13]. Although the authors enrolled COPD patients, there was no criterion regarding smoking. It is possible that never smoker patients were enrolled in COPD group. This may have caused different result since smoking is known to increase IL-33 level [15].The mechanism of how IL-33 was involved in the pathogenesis of COPD still needs to be elucidated. Byers et al. [16] showed that respiratory viral infection in COPD patients leads to an increase in lung epithelial progenitor cells that are programmed to increase IL-33 expression. Subsequent epithelial danger signals stimulate the release of IL-33. IL-33 increases IL-13 production and results in airway mucus formation. Recently, Li and colleagues [17] showed that IL-33 induces production of autoantibodies against respiratory epithelial cells, which can be a potential mechanism for inflammation and alveolar destruction in COPD.

There have been no reports regarding the association between IL-33 and COPD exacerbation. To the best of our knowledge, this is the first study showing association between IL-33 and COPD exacerbation. Moreover, all previous studies regarding IL-33 in COPD patients were cross-sectional. For the first time, we have shown that the plasma level of IL-33 is associated with future exacerbation in a prospective study. In clinical practice, the prediction of the risk of exacerbation is important. Although several factors are known to be associated with the risk of exacerbation, they cannot perfectly predict the exacerbation. The association between IL-33 and COPD exacerbation is novel and will contribute to improve the ability to predict the risk of exacerbation.

Currently, there is not established mechanism of how IL-33 is involved in the pathogenesis of acute exacerbation in COPD. A previous study showed that smoke alters the lung microenvironment in COPD. Epithelial-derived IL-33 was upregulated by smoking and the distribution of ST2 (the IL-33 receptor) was altered. IL-33 drives an exacerbated Th-1-cell-like inflammatory response to viral infection [15]. Viral infection is a major cause of acute exacerbation and subsequent exaggerated inflammation is a key factor in the pathogenesis of exacerbation. Since anti-IL-33 is under development, in the future, studies on whether anti-IL-33 can mitigate acute exacerbation in COPD are needed.

There are several limitations in this study. First, the number of patients enrolled patients with COPD was relatively small (n = 62). However, all enrolled patients met strict inclusion criteria including a history of smoking greater or equal to 10 pack-years. Moreover, all enrolled patients were managed by an airway specialist in a tertiary referral hospital. Also, patients were prospectively followed up and monitored for the occurrence of acute exacerbation. Second, male predominance is another limitation. This is due to very low smoking rate in female in Korea. Thus, the result of this study may not be generalized. However, there has been no evidence that the role of IL-33 differs between males and females. Third, Lung tissue was obtained in patients with lung cancer. There may be a bias from the control group. The control group in this study may not represent normal non-smokers because they have lung cancer. Fourth, the plasma IL-33 was not measured in control patients. However, the comparison of blood IL-33 level between COPD and control was already reported in previous study [6]. Despite this limitation, only a few studies have analyzed the expression of IL-33 in human lung tissue.

## Conclusion

The plasma level of IL-33 in patients with COPD was significantly associated with the risk of exacerbation in prospective follow up. The expression of IL-33 was higher in COPD patients compared to control patients. Expression of IL-33 was positively correlated with smoking pack years and negatively correlated with FEV_1_ (%). Further studies regarding the role of IL-33, especially in exacerbation, are needed.

## Data Availability

The datasets used and/or analyzed during the current study are available from the corresponding author on reasonable request.

## Abbreviations

ANOVA: Analysis of variance
BMI: Body mass index
CAT: COPD assessment test
CI: Confidence interval
COPD: Chronic obstructive pulmonary disease
CS: Cigarette smoke
ELISA: Enzyme-linked immunosorbent assay
FeNO: Fractional exhaled nitric oxide
FEV_1_: Forced expiratory volume in 1 s
FVC: Forced vital capacity
GOLD: Global initiative for chronic obstructive lung disease
H&E: Hematoxylin eosin
IL: Interleukin
ILC2: Innate lymphoid cells
IRR: Incidence rata ratio
mMRC: Modified medical research council
OD: Optical density
PFT: Pulmonary function test
