# Correction to: Association between plasma interleukin-33 level and acute exacerbation of chronic obstructive pulmonary disease

**DOI:** 10.1186/s12890-021-01703-3

**Published:** 2021-10-29

**Authors:** Hyonsoo Joo, Seoung Ju Park, Kyung Hoon Min, Chin Kook Rhee

**Affiliations:** 1 Division of Pulmonary, Allergy and Critical Care Medicine, Department of Internal Medicine, Uijeongbu St. Mary’s Hospital, College of Medicine, The Catholic University of Korea, Seoul, Republic of Korea; 2 Division of Pulmonary, Allergy and Critical Care Medicine, Department of Internal Medicine, Jeonbuk National University Hospital, Jeonbuk National University Medical School, Jeonju, Republic of Korea; 3 Division of Pulmonary, Allergy, and Critical Care Medicine, Department of Internal Medicine, Korea University Guro Hospital, Korea University College of Medicine, 148, Gurodong-ro, Guro-gu, Seoul, 08308 Republic of Korea; 4 Division of Pulmonary, Allergy and Critical Care Medicine, Department of Internal Medicine, Seoul St. Mary’s Hospital, College of Medicine, The Catholic University of Korea, 222 Banpodaero, Seochogu, Seoul, 06591 Republic of Korea

## Correction to: BMC Pulm Med (2021) 21:86 https://doi.org/10.1186/s12890-021-01423-8

Following publication of the article [1], it came to the authors’ attention that incorrect R values had been provided for Fig. 4a, b.

**Fig. 4:**
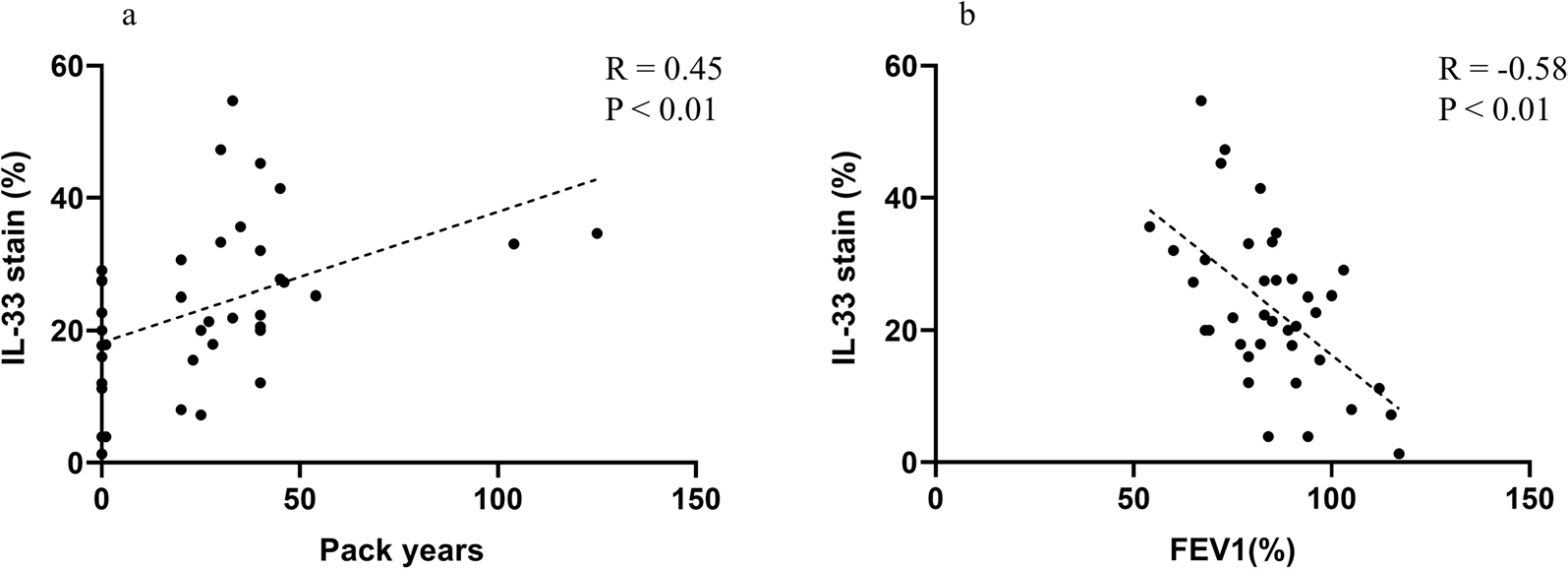
Correlation between the expression of IL-33 in lung tissue and amount of smoking (**a**). Correlation between IL-33 and FEV_1_ (%) (**b**). Abbreviations: R, Pearson correlation coefficient; IL, interleukin; FEV_1_, forced expiratory volume in 1 s

The figure has been updated in the published article and the correct values may be found in this correction article.

The authors thank you for reading and apologize for any inconvenience caused.
